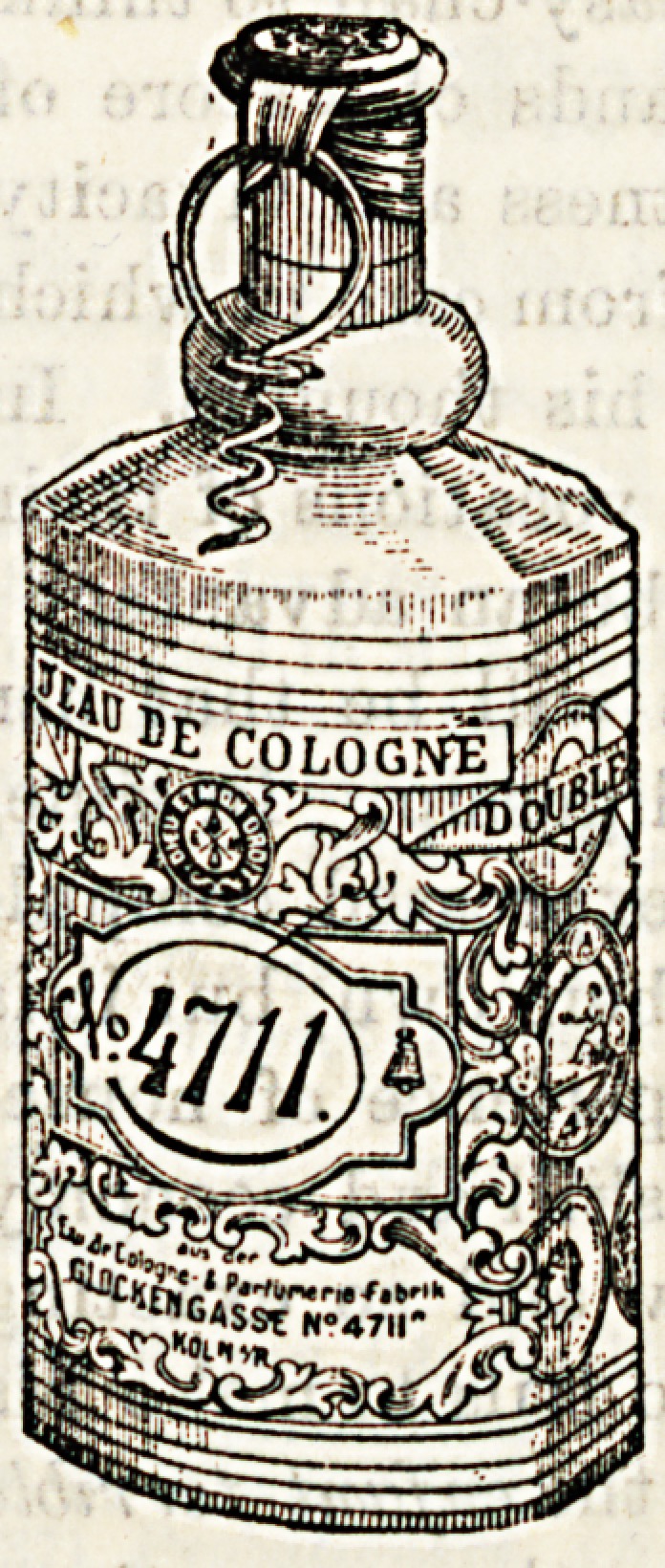# The Hospital Nursing Supplement

**Published:** 1894-11-17

**Authors:** 


### "The Hospital" Nursing Mirror.

Being the Extra Nursing Supplement of "The Hospital" Newspaper.

[Contributions for this Supplement should be addressed to the Editor, The Hospital, 428, Strand, London, W.C., and should have the word "Nursing" plainly written in left-hand top corner of the envelope.]

### News from the Nursing World.

### STUDENTS OR NURSES?

Granting the possibility of a student proving a good nurse, or a nurse becoming a fair student, there is obviously some danger of probationers soon considering "cases" of primary and patients of secondary importance. This tendency is evinced by an argument that three months' preliminary training will fit young women to benefit by the clinical teaching given in hospital wards to the students. For the latter, two or three years' preparation is considered necessary ere they receive bedside instruction. When they bring their theoretic knowledge to be thus practically supplemented by experience of dressings and by general personal acquaintance with the sick, they desire the assistance of intelligent skilled nurses, not women to whom a three months' course of anatomy, hygiene, and physiology has given a thirst for the science of medicine rather than for the art of nursing. Certainly candidates for training need increased preliminary instruction in the manual part of domestic work before entering on the study of the skilled handicraft of their profession, but with this practical foundation and a large-hearted knowledge of humanity, intelligent women may well relegate science to its proper place as the supplement, and not as the first essential to the development of a true nurse.

### OUR CHRISTMAS COMPETITION.

We are drawing so near to Christmas that the subject of our needlework competition becomes one of pressing importance, and we trust that every one of our readers will bear the matter in mind. Everybody can help, for even those unable to work can provide materials for others to use up profitably. There will be many sick folks in hospital wards on Christmas Day, each glad to receive a useful gift to supplement the sparse outfit which is all that their own limited resources can accomplish. The return home in winter means, to most hospital patients, exchanging warm wards and good nourishment for very inferior comforts, and woollen garments are most valuable in such circumstances. The prizes offered are: 20s. for the most serviceable dressing gown; 10s. for the best flannel shirt; 7s. 6d. for the best flannel petticoat; 7s. 6d. for the best over petticoat; 7s. 6d. for the best bed jacket; 5s. for the best knitted pair of men's socks; and 2s. 6d. for the second best pair. We hope those who do not care to compete will contribute useful garments of all descriptions, and send them to 428, Strand, by December 18th.

### ONLY HUMAN LIVES!

Last week a clergyman, one of the Kidderminster Guardians, supplemented his remarks on measles, to which we lately referred, by some original statements on typhoid, of which he says, according to the local press, "If he had a case in his own house, he should never dream of calling in two trained nurses to look after the patient, nor would any other person with ordinary common sense. . . . It was a disease which arose from filth, and was not transmitted if reasonable care were taken. The disease was not in the strict sense of the word infectious." Fifty sick persons in Kidderminster Workhouse are in the hands of three untrained women who are assisted by paupers. While one of the latter was left in charge of a case of measles the child was nursed at an open window, eventually becoming so ill that the medical officer feared it would die. He therefore secured a trained nurse, whose presence roused the wrath of the Rev. W. Finch. Mr. Stretton, the medical officer, asked, "Why should the children of the house be exposed to death, when by the arrangements he suggested and by skilled nursing their lives might be saved, and they might be spared having illness altogether?" In commenting on these courageous utterances, a Guardian remarked that "The doctor did not calculate the cost; he did not care a button about the expense. He only (!) tried to save life, cost what it might." Another Guardian affirmed that he would not mind risking a charge of manslaughter in case an inmate died "and it was assumed that death was due to not engaging a professional nurse. He was not afraid of any inquiry which the Local Government Board might make."

### RUSHDEN.

Miss Peter, Inspector of the Queen's Jubilee Institute, was present at the fourth annual meeting of the Rushden Nursing Association. The chair was taken by the Rector, the Rev. C. J. Gordon. The year just expired was the third and last for which a grant had been promised by the Institute; but it has been resolved by the authorities to give £20 next year and £10 the following one, on account of the Nursing Association greatly needing help. The work at Rushden appears to be thoroughly well done and much valued in the district.

### GOOD WORK APPRECIATED.

A charming tribute was recently paid to the valuable work done by Miss Rebecca Horne at the Cottage Hospital at Moreton-in-Marsh, where she holds the post of matron. Eleven hundred and thirty persons subscribed to the gold watch and chain, accompanied by a cheque, which was presented to her "in memory of twenty-one years of devoted self-sacrifice." A large gathering of friends and neighbours took place on the occasion, and several pleasing speeches were made, showing the esteem and affection with which Miss Horne is regarded in the whole district.

### DISTRICT NURSING AT RUGBY.

The Rugby District Nursing Association (affiliated with the Queen's Jubilee Institute) continues to work most satisfactorily, and the services of Nurse Reid are appreciated by committee and patients. The thanks of the fifth annual meeting were given to Miss McClure for assistance rendered while holding office as lady superintendent of the association. The Hon. Mrs. Tower was elected to this post during the ensuing year, and Dr. Percival was re-elected President.

### THE FIFTH TIME OF ASKING.

The Local Government Board recommends that six nurses be added to the staff at the Dudley Workhouse, and it is asserted that this is the fifth time a similar piece of advice has been given to the Guardians, and that "four times it had been resented" and not followed. A similar policy might have prevailed on the fifth occasion, had not the medical officer been in favour of two new nurses, although he considered that during his twenty-three years in office the institution had been "particularly free from accidents." The appointment of the two nurses was finally agreed to, apparently merely to avoid awkwardness should anything happen through this warning being disregarded. The duty of providing adequate nursing for sick paupers does not seem yet acknowledged by the Dudley Board.

### ACTIVITY AT BELFAST.

Last year the Sunshine Society began its existence at Belfast with two members and now it has fifty, including the Marchioness of Londonderry and the Countess of Shaftesbury. The report says: "The Sunshine Society aims to brighten the lives of the sick and the poor in our midst after our hospitals and nurses have done all possible by their work of healing." Days in the country or at the seaside are provided during the summer for numerous parties of two convalescents, the fare being defrayed for them and a dinner basket provided. It is pleasant to think of the invalid whose first acquaintance with a picnic is probably thus made, investigating the provision prepared by kindly hands. Weeks at the seaside are also granted by the society, which has already proved itself an invaluable auxiliary to the work of the district nurses. Increased funds are needed to enable the Nursing Association to provide a greater number of nurses for the sick poor in their homes in the growing city of Belfast.

### INCREASED ACCOMMODATION REQUIRED.

With small-pox making such a claim on the resources of the Cork Street Fever Hospital, Dublin, additional nurses' quarters are urgently needed. The obvious necessity for those in attendance on small-pox cases, whether nurses or servants, being strictly isolated from the rest of the staff, explains the need for providing a separate block with as little delay as possible. An appeal for funds has been issued, the expenditure of the institution being largely in excess of its income.

### PROVISION FOR SICKNESS.

The organisation of a "Sick Nurses' Benefit Fund" forms the subject of an article by Miss MacDonnell in the November number of our American contemporary, *The Trained Nurse.* Our sisters in the States have hitherto displayed more inertness in the matter than might have been expected from those belonging to a progressive nation. In their anxiety to avoid involving themselves in any scheme in which they feared to find the taint of so-called charity, they have postponed the consideration of this most pressing matter. Sickness, however, cannot be ignored, and our American sisters are well advised to make provision for it now whilst they are possessed of health and youth. Although the need for a scheme is now acknowledged, all plans and suggestions remain undeveloped. There has been an increase in the number of graduates at the Boston City Hospital, and the nursing staff now amounts to ninety-six. Even with the addition of the Nightingale Nurses' Home, the accommodation is inadequate, and further structural alterations are contemplated.

### GOOD TIMES.

"I think I shall take up private nursing," said a newly-emancipated probationer. "Do you think you are suited for that kind of work?" protested one of the home circle. The girl looked surprised. "I don't know, but I met a girl the other day who said she believed that private nurses had very good times. So I'm going to try." But a short experience proved conclusively that the good of the patient is by no means always identical with "a good time" for the nurse.

### OUTWARDLY CLEAN.

"The place looked so beautifully clean" is the general remark of the unprofessional visitor who has penetrated to the wards of a workhouse infirmary, the spotlessness of the rooms being taken as a sample of the general condition of the patients. Pauper help is certainly efficient on floors, chairs, and tables, and many hours are consumed in incessant toil by the "able-bodied" in keeping the boards up to the required standard of whiteness. The scrubbers, male and female, have been all their lives used to similar work, and can do it, but in the cleanest wards the nursing may be of the scantiest. A white quilt does not prove that the draw-sheet is smooth or the poor patient's back sound, and long nights passed in loneliness and pain make the sufferer very indifferent to the praise which he hears bestowed by the master on the rough-handed wardsman who can polish a floor better than he can nurse a fellow pauper.

### SHORT ITEMS.

The little inmates of the Nursing Home for Invalid Children, Witney, Oxon, sent a gift of shoes knitted by themselves to H.R.H. the Duchess of Teck for the use of Prince Edward of Wales. They received a charming photograph of the infant prince, in company with a kindly acknowledgment of the shoes. Princess Beatrice has also given a picture of her little daughter to the home, and the Duchess of Albany has sent photographs of her children. These gifts are naturally much valued by the small invalids.—The funds of the Littleborough District Nursing Association have decreased seriously, which makes a special appeal for subscriptions necessary. A liberal response is looked for, as the value of the association is as well known as it is appreciated by the patients.—Miss Lina Cunningham is leaving Falmouth, where she has held the appointment of matron at the Hospital and Dispensary, on account of her approaching marriage.—Contributions for the sale of work at the Trained Nurses' Club, 12, Buckingham Street, should reach the secretary in the last week in November.

### French Schools for Trained Nurses: Their Origin and Organisation.

By Our Own Correspondent.

### I.—PARIS SCHOOLS.

French schools for trained nurses bear but little if any resemblance to their English congeners. The organisation has been in existence about seventeen years. The schools provide instruction only, none of them supplying board or lodging for either male or female pupils.

The Paris Schools, which are the first we propose to describe, were created in pursuance of a vote of the Municipal Council, November 25th, 1877, proposed by Dr. Bourneville, the editor of the *Progres Médical*. Fortunately for the success of these municipal schools Dr. Bourneville was elected director of them, and has made them prosper. This vote of the Municipal Council resulted in the organisation by the *Assistance Publique* of two schools for training nurses at the Bicêtre and Salpetrière Hospitals, where the aged poor of both sexes are housed and medically treated. The Salpetrière school was opened in April, 1878, the Bicêtre school in the following month, and that of the Pitié Hospital was not inaugurated until 1880.

One of the principal reasons which induced the Municipal Council to organise these training schools for nurses was that they found themselves unable to work in harmony with a staff composed of Sisters of Charity. The sisters were ignorant of the duties of well-trained nurses, but too often following a routine not based on scientific principles. Administrative difficulties also arose, for as sisters of charity are, in the same way as lay nurses, required to prove that they possess the necessary qualifications for hospital nursing, they are with difficulty recruited when required.

In two of the three municipal schools, the Bicêtre and Salpetrière, primary and secondary instruction are provided; at the Pitié school, professional teaching only.

The curriculum of studies includes two kinds of lectures—theoretical and practical. The theoretical course is divided into seven sections, as follows: (1) Hospital administration and hospital book-keeping, seven lectures; (2) elementary anatomy, six lectures; (3) elementary physiology, six lectures; (4) dressing and elementary surgery (*petite chirurgie*), eighteen lectures; (5) hygiene, twelve lectures; (6) elementary dispensing (*petite pharmacie*), ten lectures; (7) care of lying-in women and children, four lectures.

The course of practical lectures includes the medical and surgical teaching regarding the means required to ensure asepsis, and the requisite arrangements after death; how to make dressings, to take the temperature of the patients and arrange their beds, how to receive, wash, and bathe a new-born infant, and to dress the umbilical cord, to dress a baby, and to keep its cradle in order, and to manage a *couveuse* (an apparatus used for children prematurely born); likewise all details concerning suckling, and the precautions to be taken before handling new-born children.

The scholastic year commences on October 1st and ends in August. The theoretical lectures are given twice a week, from eight to nine p.m. Each professor delivers from six to eighteen lectures, and sets the subjects for two papers to be sent in by the pupils who follow his course. The practical lectures are given six or eight times a week. The pupils are divided into sections in order that the number in each class shall be limited. During the month of July the pupils in each class write a paper on every subject that has been lectured on during the scholastic year, and prizes are awarded to the best. The merit of the papers in both the theoretical and the practical classes decides the award of the *diplôme d'infirmière* (nurse's certificate). This diploma enables the holder of it to obtain the post of *surveillant* or *surveillante*, who direct the ordinary ward nurses. The latter often attend the lectures in order to gain this diploma. To defray the necessary expenses the Municipal Council annually contributes 18,400 francs (£736) to the expenses of the classes, which is spent as follows: Salaries of lecturers, 12,000 francs (£484); printing, books, and office furniture, 3,500 francs (£140); prizes, savings-bank books, and cases of instruments, 2,800 francs (£112). In addition to this allocation, 5,080 francs (£520) are yearly devoted to the maintenance of thirteen pupils who have taken prizes in the three hospital training schools.

The question will naturally occur to many minds, why these nursing schools are organised in what we should call asylums instead of in general hospitals. The answer is that the Bicêtre and Salpetrière hospitals were chosen because they possess a large staff, capable of organising and carrying out a scheme of good practical teaching. Also there are no religious superintendents in these hospitals to put obstacles in the way of lay nurses. Nevertheless, the teaching in these hospitals cannot be complete, inasmuch as there are no lying-in or surgical wards, nor any for acute diseases. The inmates are the aged poor of both sexes, suffering from chronic diseases, epilepsy, and mental affections, and likewise idiot children. In order to remedy the deficiency noted, the nursing school was organised at the Hospital La Pitié, where examples of every branch and specialty of medicine and surgery can be studied and utilised in the courses of lectures for trained nurses, and where there is a constant influx and efflux of patients. In consideration of these advantages, so necessary for the thorough education of nurses, M. Bourneville is desirous that the Hospital La Pitié should fulfil the function of a finishing school of nursing (*Ecole de Perfectionnement*). These three schools not only train the nurses of the respective hospitals enumerated, but also many outsiders; in fact, anyone desiring to attend the lectures and pass the examinations can do so.

Pupils who have no connection with the Bicêtre, Salpetrière, and La Pitié Hospitals write their names on a register *ad hoc* kept at the office of the hospital directors.

### Our American Letter.

A pretty little hospital has been recently opened at Fitchburg, Mass. It stands in about 400 acres of land, and is said to be admirably equipped. The nurses' accommodation is excellent, and it is in contemplation to organise a training school, although the wards are at present arranged for twelve patients only. The hospital is built and endowed through the generosity of the late Mr. Gardener S. Burbank, and will be known as the Burbank Hospital. The matron, Miss Elizabeth Sumner, graduated from the Waltham Training School, and was a district nurse at Philadelphia before taking charge of the Newburgh Hospital, N.Y. There are four nurses, and a house orderly and one servant who is to undertake the duties of cook, laundress, and housemaid.

There are ten medical schools for women in the U.S.A. and one in Canada, and it is asserted that nearly 2,000 ladies, including 120 who devote themselves to homœopathy, are in practice as doctors in America.

The new buildings of New Rochelle Hospital, N.Y., were opened with considerable ceremony and prove a great acquisition to the institution. Miss Scovell has resigned the matronship of the hospital at Newport, R.I., and has been succeeded by a graduate of the General Hospital, Massachusetts, Miss Lucy V. Pickett, who for five years has held the post of head nurse at Newport. Miss Ella Gilliland has become Superintendent of Nurses at Milwaukee Cottage Hospital, Waumatosa, Wisconsin.

### Nursing in Ireland.

### THE CITY OF DUBLIN HOSPITAL.

THE alterations in the City of Dublin Hospital are well nigh completed. Since its establishment more than sixty years ago the hospital has maintained an excellent position in popular estimation, being one of the most efficient in Ireland.

In 1832 three private houses, once a portion of the Royal Charter School, were utilised as a hospital for the southern suburbs of Dublin, and in that building, with the addition of a detached wing for infectious cases, has been carried on a noble struggle to meet the increasing demand upon its resources.

As may well be imagined, however, the building was very far from fulfilling modern hygienic requirements. The ceilings were low, the windows rather small, and the whole aspect of the place gloomy and depressing—all which defects were mourned over by those connected with the hospital. Owing to lack of funds better things seemed unattainable. However, Lord Pembroke's generous promise to contribute £6,000 towards the rebuilding and extension of the hospital was contingent on the fulfilment of two conditions, one being that the façade of the new building should be artistically in keeping with the architecture in the vicinity, and that the remainder of the sum required should be raised by public effort. This was happily accomplished when the great bazaar and fête in May, 1893, achieved such financial results as its promoters had not even anticipated. £12,000 remained in hand after the payment of all expenses connected with the fête, which, with Lord Pembroke's donation, sufficed to carry ont all the structural alterations. Funds, however, are still needed to provide for the maintenance of increased numbers of patients.

Only those acquainted with the hospital in former days can fully realise the transformation which has been effected. A handsome Queen Anne façade has revolutionised its outward aspect, but the interior metamorphosis is even more complete. The large airy well-lighted wards, with polished flooring and pretty fire-places, the broad corridors, and compact little kitchens are indeed improvements.

During the alterations the number of patients admitted to the hospital has been greatly limited, though work was never altogether suspended, the Drummon l Wing, usually reserved entirely for infectious cases having been utilised for ordinary work, infectious diseases being temporarily excluded. As the new wards are opened one by one the number of inmates gradually increases, and before long it is hoped all the discomfort which has been inevitable for so long may be only a memory to the medical and nursing staff, who have been the principal sufferers. The probationers, indeed, all of whom are drawn from the adjacent City of Dublin Nursing Institution, whither they repair for meals and pass the night, have suffered comparatively little, and the patients have been made as comfortable as though the regular routine had not been interfered with. But the lady superintendent and staff nurses, whose portion for a considerable time has been a perfect chaos of "what was, what is, what will be," deserve the warmest praise for their good-tempered cheerfulness under trying circumstances, and they are now beginning to reap their reward, their pretty sitting-room, already habitable, affording a bewitching haven of comfort, and a view—it is on the fourth floor—right away over roofs and chimney pots to the open country and the blue Dublin mountains.

The nurses' dining-room is not yet furnished, nor are the bed-rooms which they will eventually occupy, with the exception of one or two, and those only in very fragmentary fashion. Pretty little apartments they are, equal in number to the staff, each of whom will enjoy the luxury of a separate room, while the windows command the same magnificent view as those ot the sitting-room.

A hydraulic lift is in process of construction, and a new and improved operation theatre is contemplated when funds admit of its erection. In the basement the out-patients' dispensary is located, and kitchen and laundry work are at present carried on in temporary offices, but both departments are provided with improved appliances. So these important portions of hospital work are carried out most efficiently. Workmen are still busy about the entrance hall, lower corridors, &c., but considering that all progress was suspended for over two months owing to a bricklayers' strike, followed by the failure of the contractor originally entrusted with the alterations, it seems wonderful that still more does not remain unfinished Indeed now a very short time will serve for the finishing touches, and then accommodation will be secured for 150 patients and a staff of 30 nurses, including probationers. The City of Dublin Hospital will then compare favourably with many larger institutions in every respect save one, and that is the absence of an ambulance; but doubtless this defect will not long remain unremedied.

### Appointments.

MERCERS' HOSPITAL, DUBLIN.—Miss Eleanor J. Law has been appointed Matron of this hospital. She was trained at the London Hospital and held the post of staff nurse there, afterwards becoming Sister-in-Charge of St. John's Hospital for Diseases of the Skin, Leicester Square, where she remained for nearly three years. We congratulate Miss Law on her appointment and wish her success in her new work.

INFECTIOUS DISEASES HOSPITAL, CAMBRIDGE.—Miss Norris, whose promotion to the post of Matron of this hospital was announced last week, now writes to say that she does not desire to hold the appointment.

### Minor Appointment.

WALLSEND-ON-TYNE.—Miss M. Rudd has been appointed District Nurse by the Wallsend Sick Nursing Association. Miss Rudd has been district nurse for St. Andrew's Parish, Penrith, for the last four years, and her work there has been highly appreciated. We wish her all success and prosperity.

### Notes and Queries.

### Queries.

(36) *Wigan.*—What hospitals are there at Wigan, and what do the nurses wear?—*A Weekly Reader.*

(37) *Probationers.*—Will you kindly tell me in what general hospitals a girl of eighteen would be admitted as probationer? A friend of mine wishes to enter one.—*Nurse Bertha.*

(38) *Maternity.*—Where can training in n onthly nursing and midwifery be obtaine l in Manchester?—*Reader.*

(39) *Mental.*—Can you tell me of a handbook on brain disease which gives information on dementia, &c.?—*W. H.*

(40) *Text Book.*—Is Lewis's "Theory and Practice of Nursing" suitable for a young nurse, or Miss Hampton's book?—*Nurse Millicent.*

(41) *Charts.*—Where can I get these for private nurses?—*Midwife.*

(42) *District Nurse.*—Information wanted as to a post of this kind near Glasgow.—*Nurse H.*

(43) *Blind.*—Can you tell me of any institute for the blind in or near London?—*A. C. C.*

### Answers.

(36) *Wigan (A Weekly Reader).*—By writing to the Matron you can secure information with respect to the nurses' uniform at the Royal Albert Edward Infirmary and Dispensary, Wigan.

(37) *Probationers (Nurse Bertha).*—Eighteen is far too young for general hospital. Children's hospitals and asylums sometimes take training applicants under twenty. It would be better for your friend to wait until she is older.

(38) *Maternity (Reader).*—Write for terms and rules to Matron, St. Mary's Hospital (the Manchester and Salford Lying-in Hospital), where there are 10 nurses and 21 midwives, or to Manchester Maternity Hospital, 60, Upper Brook Street, which is in connection with the Southern Hospital.

(39) *Mental (W. H.).*—The book you want is "Outlines of Insanity," by Francis H. Walmsley, M.D. Published by the Scientific Press, 428, Strand.

(40) *Text Book (Nurse Millicent).*—Lewis's "Theory and Practice of Nursing" or "Lectures on Nursing" by Miss Lückes, are suitable for young nurses. Miss Hampton's excellent book is more advanced.

(41) *Charts (Midwife).*—Write to Scientific Press 428, Strand.

(42) *District Nurse (Nurse H.).*—Write to Inspector of Scottish Branch of Queen Victoria Jubilee Institute, Edinburgh.

(43) *Blind (A. C. C.).*—You will find full information in *Burdett's Hospital Annual*, issued by the Scientific Press, 428, Strand.

### Wants and Workers.

CAN any reader of THE HOSPITAL tell F. B. if there is any opening f a Private Nurses' Institute at Folkestone or Great Malvern?

### The American Woman at Home.

### I.—HER HOME.

A good deal has been written of recent years about the American woman, or rather, the young American girl; but chiefly as she appears in Europe, contrasting her Trans-Atlantic ideas with our conventions, her high opinion of herself with our old-fashioned notions of women's power and place. Thus viewed, she appears brilliant, independent, unconventional, as any strange thing does—as even the staidest British matron might do in the eyes of a native of Hindostan or China, as we know an ordinary English girl does to the mind of a French *jeune fille bien élevée*; therefore, to form any true notion of the American or any other woman, we must judge her in her natural surroundings—at home.

At home—when she has one; when she has not begun her married life, as so many do, in the idleness and publicity of a boarding-house. The primary cause of the popularity of the boarding-house is the difficulty of procuring competent service at a reasonable price. This operates to make the American woman of the country districts one of the most hard-working and competent of housekeepers, while her sister of the towns becomes one of the idlest. The one does her own work, or all but the roughest and most mechanical of it; the other makes her home in an hotel. Hotel life may be regarded as one of those experiments in co-operative housekeeping which on this side are constantly being recommended, but seem never to be ardently taken up. And, indeed, it shows that even if it could be made perfect on the material side, co-operative housekeeping fails on moral grounds. The human soul craves for solitude; a married couple craves, or should crave, for *solitude à deux*. Yet this is in the boarding-house unattainable except in their own apartment, and the time for intimate conversation is limited by the demands of the hap-hazard society into which they are flung. The husband cannot, as husbands sometimes wish to do, eat his dinner in silence, resting from the fatigues of the day, nor throw himself, unquestioned, into his own particular easy-chair to think out the problems of the future. The demands of a score of people round him compel him to the alertness and vivacity of his day's manner, and the very freedom from care in which his wife rejoices makes her unfit to share his thoughts. In the case of a few exceptional women with vocations of their own, the absence of domestic cares may be an advantage; but for the average woman the only result will be that her vocations will degenerate into shopping and gossip. In time, of course, a baby may exercise its loudly-expressed compulsion to drive its parents to a home of their own, but it is certainly better to have obtained some experience of household duties, the management of servants, skill and economy in marketing, and the rest, before a new and so exacting factor enters. And alas for the baby who fails to make his parents go to housekeeping! He becomes the *enfant terrible* of the place, the precocious *gourmet*, the premature philosopher, who is doomed to start in life with a crowd of shallowly-shrewd ideas, and, which is even worse, a demoralised digestion. The decay of home-life is a constant subject of lament among the more thoughtful writers of America; yet it was an American who wrote "Home, Sweet Home"!

Given the home, however, we find even there a sort of instinct of publicity. The sitting-rooms open into each other, and are separated only by portière curtains or some still slighter arrangement of hanging cords. Sliding doors, even when they exist, are rarely brought into requisition. The intention is to permit the free circulation of heat in winter and air in summer. The first is done thoroughly well; the second hardly meets English requirements. For though the mistress of the house is careful to draw down blinds and close windows during the heat of the day, she is generally less certain to open up the house after sundown. American houses are always, from the English point of view, too hot. In summer this may be inevitable, but why, in winter, should people convert their houses into ovens, making themselves so soft that when they go out into the piercing cold they can only escape a chill by donning a paralysing amount of clothes? With a trying climate, atrocious roads, and this over-sensitive and over-laden body, it is impossible for an American lady to take the exercise which we should consider necessary to health, and as a matter of fact she is infinitely more delicate than her English cousin. I hope the estimate given me by one medical man was over-stated, that not more than one American woman in ten was fitted to be a wife and mother, but certainly the number of ailing women, to whom doctoring and drugging are a matter of necessity, is infinitely greater among them than among us.

In one way the indifference to publicity which seems to me the keynote of American life works very charmingly; I mean in the exterior of the houses. There neither wall, hedge, nor railing arise to separate one man's garden from his neighbour's, and as the gardens are usually ample and well-kept, and the houses picturesque and varied in architecture and material, the aspect of an American street of the better class is that of a well-kept park, with handsome buildings dotted about at irregular intervals. Brick and stone are the more expensive, and therefore more highly valued, building materials, but some of the wooden or "frame" houses, as they are called, are as attractive in appearance, and the only objection to them is their greater liability to fire. When on a summer evening one sees a family sitting on the verandah of their house, framed by luxuriant creepers, while in the gloaming the fire-flies flash and glimmer on the lawn, one receives one of the pleasantest impressions of American life.

Inside the house a great deal is made of labour-saving inventions; but for my own part I do not think that our English arrangements are so far surpassed as we in our innocence believe. Some suggestions, indeed, we might borrow, but we could easily repay them in kind. And in an American household the work is hard—much harder for each servant than here. The general fault of the good American housekeeper is that she tries to keep up a certain style of living with about half the number of servants necessary—from our point of view—to attain it. This does not arise either from poverty or economy; these very ladies spend most lavishly on all the appointments of their table and the decorations of their rooms, although, according to the testimony of the Columbian Association of Housekeepers, they are often stingy in the equipment of their kitchen and laundry; but from the acknowledged difficulty of obtaining good servants. Irish maids are dirty and untidy, Germans are heavy and ignorant, negroes are dishonest and lazy, and French Canadians are attacked with nostalgia, and wish to go home as soon as they have learned your ways; while the American girl will rather take three dollars a week in a store than four or five dollars and her board in a kitchen. "Why can't they see that it's service in either case, and the one is as honourable as the other?" exclaimed one lady, discussing the matter with me. And the result is that the service in American houses is often bad; that if it is good it is only through the exertions of the mistress, which leave her fagged, nervous, and irritable; that hardly anyone keeps as many of these domestic pests as their income justifies or the style in which they live demands, and that many people do without the thing miscalled "help" altogether.

### Marriage.

Miss Hey, who was Nurse-Matron of Sir Titus Salt's Hospital, at Shipley, for two and a-half years, was married on the 10th inst. to Mr. John Norton, of Cardiff. Many kind wishes were expressed, and much interest displayed in the wedding, which took place at St Paul's Church.

### Dress and Uniforms.

By a Matron and Superintendent of Nurses.

### Nurses' Wallets.

Few firms have been more successful than Messrs. W. H. Bailey and Sons, of Oxford-street, in their provisions for a nurse's requirements. They have lately brought out an admirable wallet, which we feel sure will meet with the appreciation it deserves at the hands of our readers. The superiority claimed for this wallet over all others of the kind lies in its being dust-proof. An ingeniously-constructed

The "Hard-wear" Wallet.

The "Dust-proof" Wallet.

flap folds over in such a manner as to render the entry of dust less easy, an important consideration where surgical instruments are concerned. It hooks on to the waistband by an improved catch which prevents it slipping up, and is attached to the catch by straps which can be removed at will. The convenience of this arrangement when the wearer desires to use the wallet as a pocket-case can be readily imagined. The price in best black morocco is 10s. 6d., which is not expensive when the excellent finish and quality of the workmanship is considered. We give an illustration of the wallet as it appears when open, showing the flap which is its special feature. Messrs. Bailey also supply a cheaper wallet, called the "Hard Wear," at 3s. 6d., which is especially adapted for ward requirements, and is reasonable enough in cost to tempt a trial at any rate. Messrs. Bailey also supply the wallets fitted at moderate prices.

### Knitted Clothing.

The Knitted Corset and Clothing Company (118, Mansfield Road, Nottingham) are to be congratulated on the excellent quality of the knitted clothing which they supply direct to the public at very moderate prices. Cardigan petticoats and vests are kept in all sizes and thicknesses, and are likely to be in demand as winter approaches. Especially delightful are the pantaloons, which will be found invaluable for mountain-climbing, hunting, or skating, &c. They end at the knee or ankle, but can be had to include the foot as well. The night-dresses are supplied in either pink, natural or white wool, and will be found particularly adapted to all who suffer from rheumatism or a bad circulation. The knitted corset, however, is the specialité for which this firm is noted; it is made in silk, cotton, or wool, as desired, and fits comfortably to the figure without unduly compressing it. For those who require support, bones or flexible steels are inserted. Endless in their variety are the knitted gloves, stockings, mittens, travelling capes, &c., all of which can be seen on application, and few articles make more useful or attractive Christmas presents than these. A cardigan jacket, with long sleeves, at 10s. 9d. would be the very thing to wear in cold weather under a cloak, and thus warmth and comfort would be effectually secured. A comprehensive catalogue and samples of material will be sent on application.

### Specialities in Ward Shoes.

Messrs. Manfield and Sons, of Bloomfield House (85, London Wall), are showing some excellent samples of ward shoes. They are made in three qualities at 4s. 11d., 6s. 11d., and 8s. 11d. respectively. The latter is a very superior article, soft and comfortable to the foot, well finished off, the uppers being of real glacé kid, all handsewn. These shoes are provided with a square military heel, capped with india rubber, and the sole is broad and well proportioned to the shape of the foot. A strap across the instep ensures the support which is so necessary to workers in a hospital. This shoe has only to be known to secure a wide-spread popularity, and the manufacturers, from the number and convenience of their branch establishments, place every facility in the way of customers. There are depôts at most of the principal towns in England, and also Paris, while in London there are no less than five, viz., 2, Ludgate Hill, 376, Strand, 307, High Holborn, 228, Piccadilly, and 13, Borough High Street.

### A Famous Scent.

Few scents are more delightful and refreshing than really good Eau de Cologne. The difficulty which exists in this country of obtaining the genuine article is at length overcome by Mr. R. J. Reuter, who, in handsome premises at 62, New Bond Street, has established a depôt for Herr Mühlen's famous No. 4711. We venture to predict that few who pass this fascinating window, wherein is displayed with so much elegance and variety flasks and flagons of artistic shape daintily tied with ribbons, will have strength of mind to resist a purchase. Apart from the delicious nature and quality of the perfume, this Eau de Cologne is possessed of considerable medicinal virtue. A few drops on cotton wool are said to allay the pain of toothache, and sprinkled on a handkerchief to be a specific for headache. As a cooling lotion for the back in bedridden cases, and a consequent preventative of bedsores, it cannot be too highly extolled. It is also invaluable as a deodoriser. In the sick room a few drops on a shovel of burning embers will be found to emit the most delightful fragrance, effectually replacing all unpleasant odour. A little added to the bath is both refreshing and invigorating, and it can also be recommended as an effectual mouth wash.

A leading physician says, "For medical purposes I always use gold label 4711. As the perfume is delicate, lasting, and pure, leaves no faint, unpleasant smell after a few hours, which inferior perfumes—Eau de Cologne especially—always do." Six bottles daintily arranged in a case for 12s. 6d. would make a charming Christmas or New Year's present, and one which could not fail to be appreciated. A sample bottle will be sent post free to any address on receipt of 2s. 6d.

### Where to Go.

Tuesday, November 20th.—Miss C. J. Wood will lecture on "The Nursing of Sick Children," at 3 p.m., at 12, Buckingham Street, Strand. Ticket for single lecture, 1s. 6d.

St. George's Vestry Hall, Little Russell Street, Bloomsbury, November 21st, at 8.30 p.m.—M Felix Volkhovsky, Russian exile, on "How I Escaped to Freedom."

For Book World for Women and Nurses, and Reading to the Sick, see overleaf.

### The Book World for Women and Nurses.

[We invite Correspondence, Criticism, Enquiries, and Notes on Books likely to interest Women and Nurses. Address, Editor, THE HOSPITAL (Nurses' Book World), 428, Strand, W.C.]

MAD SIR UCHTRED OF THE HILLS. By R. E. CROCKETT. (London: Fisher Unwin. 1894.)

Mr. Crockett's name is known to many in association with his previous works, "The Raiders," "The Stickit Minister," &c. "Mad Sir Uchtred" has a lesser claim on our attention, in so far as its bulk and length go—if literature is to be measured, that is, by the standard of the inch tape. The book before us is weird, fantastic, imaginative, full of the well known characteristics of its writer, whose aim has ever been to harmonise a story with its telling. In this case it is, perhaps, more the telling which attracts us than the tale itself. "Mad Sir Uchtred of the Hills" is really little else, to put it shortly, than the history of a curse. We are told in the opening pages that in the day of Scotland's skaith (misery) Uchtred of Garthland forgot the blessed covenants that his father had sworn. It was a common thing to forget them in those days, Kings set the example, "but God so ordained it that none who forgot them prospered. Now Uchtred of Garthland was worse than any," so it goes on to explain, "the man-beast sat in the hars of the wolf's stock"; and the man-beast, as they called the mad Laird of Garthland, was hunted of dogs and man. In the fastness of the mountains, in the dales and dells, plenty of hiding-places presented themselves, and be sure the madman hid there, well and long. It is in this hiding in these Scottish hills that the description does not read in any very attractive way. "His legs and arms were seamed and scarred—shrunk to sinew and shank bone. The frost had opened cracks in them. The dews of night clogged his hair. The red earth of his den in the wolf's stock was caked hard upon him." For his food he chased the swift mountain hare and caught it. He eat his prey quick and quivering, and lay down on the rock to sleep like the beast that he was. He that had been Sir Uchtred of Garthland and lived daintily, loving dalliance upon the cushions of Whitehall! And three years this was so, at the expiration of which period there came a happy termination—happy and unexpected. The character of Philippa, the gentle and deserted wife, who stood loyal through much temptation, is charmingly delineated, and is at once artistic and touching in its contrast with her hapless mate.

DIET AND COOKERY FOR COMMON AILMENTS. By A FELLOW OF THE ROYAL COLLEGE OF PHYSICIANS and PHYLLIS BROWNE. (Published by Cassell and Co. Price 5s.)

This little volume contains 298 pages, a goodly proportion being devoted to recipes. Some of the remarks on digestion are very practical, for the authors seem to realise the absurdity of laying down hard and fast rules for people of different constitutions, so we find "It is effort, not exercise, which is harmful; and the relation between effort and exercise is so much a matter of habit, that we shall not be surprised to observe accounts of work performed without effort by the one whose habit is work, which the unaccustomed could not accomplish without severe and sustained endeavour. . . . Clearly that which would disturb the digestion in the latter case, would leave it uninfluenced in the former." As regards the dietary which the writers consider sufficient, opinions may well differ; three meals a day, the last at half-past six to seven, being suited to those who work only with their hands, whereas people who are not very robust, or who work chiefly with their brains, will object to such restricted fare. Long intervals without food are not popular on this side the Atlantic, although our American cousins consider three meals a day sufficient in sickness or health. There is not much that is new in the pages devoted to vegetables, and the condemnation of fruit as an article of diet is somewhat too general to be acceptable. Not only are delicate persons to be restricted to oranges and grapes, but we read, "and even in health we hold the dessert to be, at the least, unnecessary." With regard to the nourishment of babies, the authors give a useful hint in saying that when milk, modified in various ways, cannot be digested, "the treatment should be in the hands of a doctor." Some of the recipes are for, indeed, dainty dishes, though the intermixing of these with the diseases savours more of popular than professional handling. The book has essentially a "popular" air within and without, and will be acceptable to those who like to lay down strict lines of diet for themselves during real or imaginary ailments.

### For Reading to the Sick

CHRIST'S SUFFERING.

**Motto.**

THROUGH the shadow of an agony cometh redemption.
—*H. H. E.*

Hence comes it that by suffering they are taught.—*Virgil.*

**Verses.**

Like a long forgotten strain
Comes sweeping o'er the heart forlorn,
What sunshine hours had taught in vain—
Of Jesus suffering shame and scorn,
As in all lowly hearts he suffers still.—*Keble.*

Count each affliction, whether light or grave,
God's messenger sent down to thee; do thou
With courtesy receive him; rise and bow;
And, ere his shadow pass thy threshold, crave
Permission first his heavenly feet to lave;
Then lay before him all thou hast; allow
No cloud of passion to ursurp thy brow,
Or mar thy hospitality; no wave
Of mortal tumult to obliterate
The soul's marmoreal calmness: Grief should be,
Like joy, majestic, equable, sedate;
Confirming, cleansing, raising, making free;
Strong to consume small troubles; to commend
Great thoughts, grave thoughts, thoughts lasting to the end.
*Aubrey de Vere.*

Oh, never is the path we tread
So drear, but if we upward gaze
The favouring smiles of Heaven will shed
Some solace for our darkest days.—*Beech.*

Lord, who hast suffered all for me,

My peace and pardon to secure,

The lighter cross I bear for Thee

Help me with patience to endure.—*Cowper.*

**Reading**

It is in submission to Love, not in striving to understand and to measure its workings, that we enter into our Lord's mind, and follow His example . . . The divine charity reveals itself in suffering, can reveal itself perfectly to us only in suffering. We are suffering ourselves; we see suffering all about us. If God had not met us and held converse with us in suffering, we must be strangers to Him. The Passion does not want theoretical explanations. It explains itself to the hungry man by his hunger, to the sick man by his sickness, to the man who is suffering from the unkindness of others by that bitterness; . . . . to him whom the accumulated sorrows of humanity are crushing by the death-anguish he has to bear. Every one feels that the Son of Man is entering into his grief—knowing the inward source of it, penetrated by the sense of it, as he never can be. All that we feel weakly, imperfectly—all that we wish to feel and do not—we are sure was part of His sympathy and agony.—*F. D. Maurice.*

Nothing but the Infinite pity is sufficient for the infinite pathos of human life.—*John Inglesant.*

God gives grace to bear all that He sees fit to lay upon us. In our sorest distresses He draws near to comfort and help us. We bear silently from God that which, if it were from the hand of man, we should rebel at and faint under. In the depth of the heart's bitterness we cling to God, "Who only knows, Who alone can comfort."—"*Heart Musings.*"

## Figures and Tables

**Figure f1:**
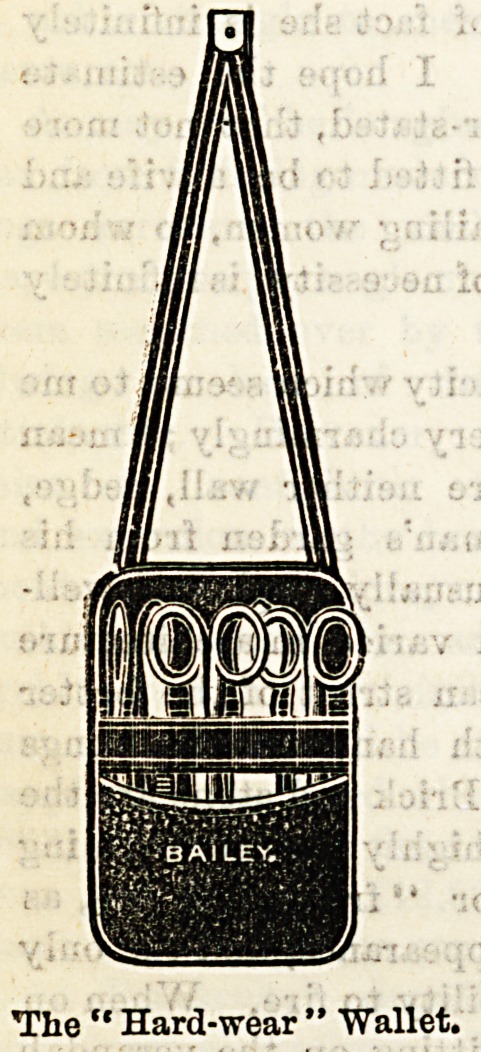


**Figure f2:**
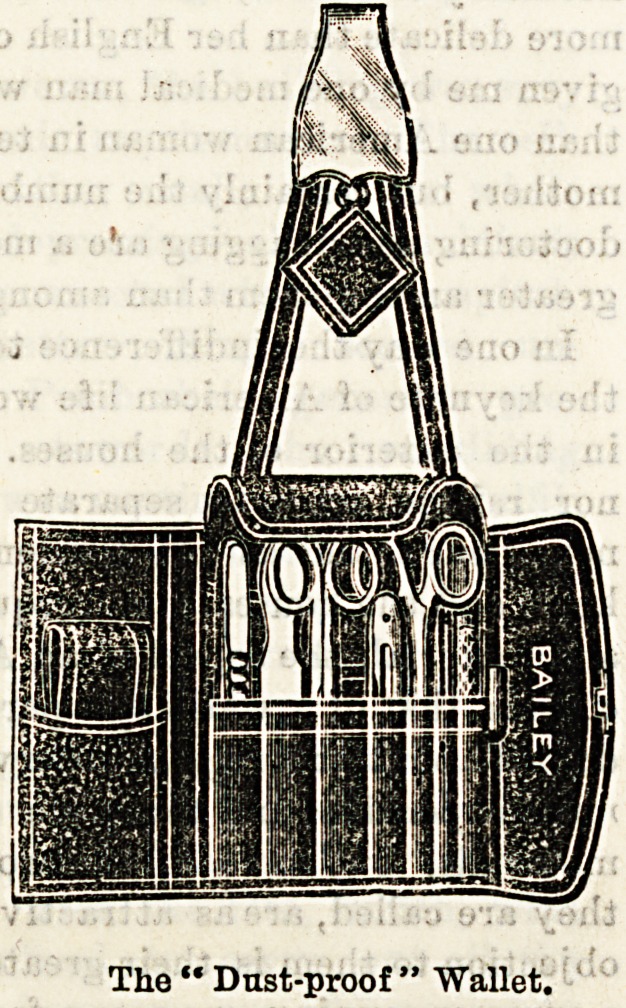


**Figure f3:**